# Reply to Sleutel and Remaut, ^“^Structural insights into *Escherichia coli* CsgA amyloid fibril assembly revisited”

**DOI:** 10.1128/mbio.01106-25

**Published:** 2025-07-11

**Authors:** Fan Bu, Derek R. Dee, Bin Liu

**Affiliations:** 1 The Hormel Institute, University of Minnesota 70188 , Austin, Minnesota, USA; 2 Department of Pharmacology, University of Minnesota Medical School 12269, Minneapolis, Minnesota, USA; 3 Faculty of Land and Food Systems, The University of British Columbia 117198, Vancouver, BC, Canada; University of Michigan-Ann Arbor, Ann Arbor, Michigan, USA

## REPLY

The discovery of functional amyloids in bacteria dates back several decades, and our understanding of the *Escherichia coli* curli biogenesis system has steadily evolved over time. Since Chapman’s seminal work in 2002, which involved isolation, characterization, and depolymerization of the major curli subunit CsgA, a variety of structural tools have been employed to elucidate curli architecture. Notably, later on, both solid-state nuclear magnetic resonance spectroscopy (NMR) and X-ray diffraction offered critical insights into the structure of *E. coli* CsgA fibrils, revealing their hallmark cross-β architecture (1, 2). Despite these advances, curli fibers have remained challenging to characterize structurally, primarily due to the high aggregation propensity and intrinsically disordered nature of monomeric CsgA.

### Structural insights into *E. coli* CsgA and *P. korlensis* R15.5 amyloid fibril assembly

Motivated by recent progress in cryo-electron microscopy (cryo-EM), we initiated efforts in 2022 to determine the high-resolution structure of *E. coli* CsgA amyloid fibrils. However, the disordered nature of wild-type CsgA posed significant obstacles for downstream cryo-EM analysis. In the course of our work, we encountered an innovative study by Chapman’s group (3), which described the engineering of a double-cysteine mutant of CsgA, with mutations placed in non-amyloidogenic regions. They demonstrated that “ CsgA_CC_+TCEP formed fibril structures of identical morphology to wild-type fibers,” as confirmed by transmission electron microscopy.

We found this mutant particularly advantageous for our study, as it enabled more controlled polymerization of CsgA. In addition, the engineered cysteines introduced two extra negative charges at pH levels above 8, potentially enhancing electrostatic repulsion and promoting fibril dispersion. To optimize structural analysis, we examined CsgA fibril morphology across a broad spectrum of buffer conditions. These efforts culminated in the acquisition of a cryo-EM density map that we were able to analyze. The resulting map revealed discernible secondary structure features of the CsgA fibrils and, for the first time, allowed us to measure the β-helical dimensions of CsgA (4), which we compared with previous computational predictions. Notably, these measurements aligned closely with the model proposed by Tian et al. (5), providing strong experimental validation.

Additionally, our lower-resolution maps revealed how two fibrils make contact or bundle together, offering further insight into their supramolecular organization. However, our study also highlighted persistent challenges: “the unambiguous determination of monomer connectivity was not feasible at the current resolution” and “the detailed interactions between molecules within a fibril remain to be elucidated through future higher-resolution structural studies.”

During the submission of our manuscript, we became aware that Sleutel et al. (6) were also pursuing cryo-EM-based structural studies of CsgA amyloids. They subsequently published a study on fibrils of *Pontibacter korlensis* R15.5, a CsgA homolog. We were excited to see that both groups independently addressed the structural question of CsgA using similar cryo-EM approaches. Given that Sleutel et al. are well-established in the amyloid field, particularly through their prior work using *in situ* nanoscopic imaging to probe *E. coli* CsgA fibrils at the single-fiber level, we appreciated the shared momentum in this area of research.

Following the publication of both studies, Sleutel and Remaut expressed concerns that aspects of our study—specifically the proposed monomer connectivity model—could be misleading. Their primary critique centered on the way we provided our CsgA model. However, we believe we were clear in presenting this model as a tentative interpretation, based on the resolution available at the time which had the highest model-to-map fitness. Throughout our paper, we explicitly stated the uncertainty surrounding monomer connectivity and employed careful, appropriately cautious language to indicate that the model was hypothetical rather than definitive. Moreover, during data processing, we applied only C1 symmetry to preserve the natural heterogeneity of the particles and avoid introducing assumptions about structural organization. We intentionally did not apply helical reconstruction or any symmetrical constraints that could artificially enforce a particular model. It is important to note that the focus of our study was never on resolving monomer connectivity, but rather on characterizing the fibril morphology and overall architecture of CsgA fibrils.

Importantly, in both their earlier paper (6) and subsequent comments, they acknowledged that their *P. korlensis* R15.5 density map suffered from the same technical limitations as ours. The Protein Data Bank (PDB) file uploaded for R15.5 (PDB: 8C50) also shows a specific assembly (head-to-tail) that is not based on an unambiguous assignment from the cryo-EM data, but rather by manually docking the AF2 predicted structure into the map. The main distinction is that they employed additional experimental techniques, atomic force microscopy (AFM) and disulfide quantification, to support their conclusion that a "head-to-tail" monomer arrangement was most consistent with their data. They also conceded that other forms of connectivity, such as head-to-head or tail-to-tail, might be computationally plausible but argued that these configurations did not exist experimentally based on their two assays and their previous study.

However, we remain cautious in interpreting these assays as definitive evidence against alternative models of CsgA assembly. For instance, their disulfide quantification results suggest that R15.5 likely forms a 2_₁_ screw axis through head-to-tail assembly, but less likely forms a purely translational head-to-tail arrangement, consistent with their AFM polarity measurements. However, they did not incorporate mutants to directly test whether R15.5 could also form head-to-head or tail-to-tail interactions. As such, we believe this assay alone cannot conclusively assert the orientation of *E. coli* CsgA assembly.

Measuring the growth of individual CsgA fibrils on a mica surface over time, using AFM, revealed that the fibrils grow much faster from the leading end compared to the trailing end (7). Furthermore, Sleutel et al. (7) found that the growth of single fibrils occurred in steps of high rates (burst phases) randomly interspersed with steps of slow growth (pauses). This could be interpreted as indicating that polymerization in the CsgA connected from N terminus to C terminus (NC-NC) orientation is faster and that polymerization in the other orientations does occur, but is much slower, leading to slow growth on the trailing end and the apparent pauses on the leading end. These observations are consistent with a mixed topology; however, they would suggest that there is a higher-than-expected number of head-head and tail-tail interactions present in the CsgA model (PDB: 8ENQ). Note that the AFM data were collected in 10 mM MES buffer at pH 6, whereas our cryo-EM data were collected in 10 mM CAPS buffer with 0.01% Tween 20, pH 10.4, and these conditions may change the overall kinetics.

Additional insights into CsgA interactions within fibrils could be gained by labeling the N- or C-terminus of CsgA with nanogold particles, as suggested and interpreted by Sleutel and Remaut, referencing the study by Chen et al. (8). However, we note that the study by Chen et al. (8) does not appear to have been designed to address fibril topology, as this work was done using a “CsgA_His_” construct that had two His_6_ tags, one on the N-terminal end before R1 and one after R5, so it is not possible to ascertain how the labeling patterns relate to CsgA connectivity.

To be fair, our paper did not deny that a head-to-tail configuration may be the most favorable mode of assembly for CsgA amyloids. However, in the interest of scientific rigor, we believe it is premature to definitively rule out alternative configurations, such as tail-to-tail or head-to-head, based on the current cryo-EM data. As Sleutel et al. (6) themselves acknowledged, their density map does not provide conclusive evidence to exclude all other connectivity models. Therefore, while we respect their methodological rigor and their significant contributions to this field, we strongly feel additional experiments are needed to fully resolve these ambiguities in the CsgA assembly model.

### Details on CsgA connectivity and assembly mechanism into fibrils remain unclear

Given the resolution limitations inherent in both studies, we advocate for an open-minded interpretation of CsgA fibril assembly, pending the availability of higher-resolution structures that can resolve monomer orientation with greater certainty. Tian et al. (5) also remark in the conclusions when modeling the CsgA monomer structure: “Finally, we note that although the amino acid sequences have provided a clear signal of the structure of individual subunits of CsgA, we have not been able to find any equally strong sequence signals to provide information about interactions between individual structural subunits. Since the function of the molecule is linked to its fibrillar state, the lack of such a signal is surprising, possibly indicating that contact specificity at the interface is reduced compared to that internally in the monomer.” Given that either R1 or R5 of CsgA can independently form fibrils (1), it is plausible that R1-R1 (N-N) and R5-R5 (C-C) interactions occur, supporting the possibility of mixed connectivity within CsgA fibrils.

In fact, we believe there are many questions that need to be further explored, such as the handedness of CsgA monomers, the detailed sidechain information of CsgA, the connectivity of CsgA monomers in fibrils, and the atomic interaction between two CsgA molecules. Perhaps cryo-EM alone would not be sufficient in solving all those questions. More techniques could be adopted to assist in solving this sophisticated problem.

### Novel methods to address CsgA fibril topology

**Fig 1:**
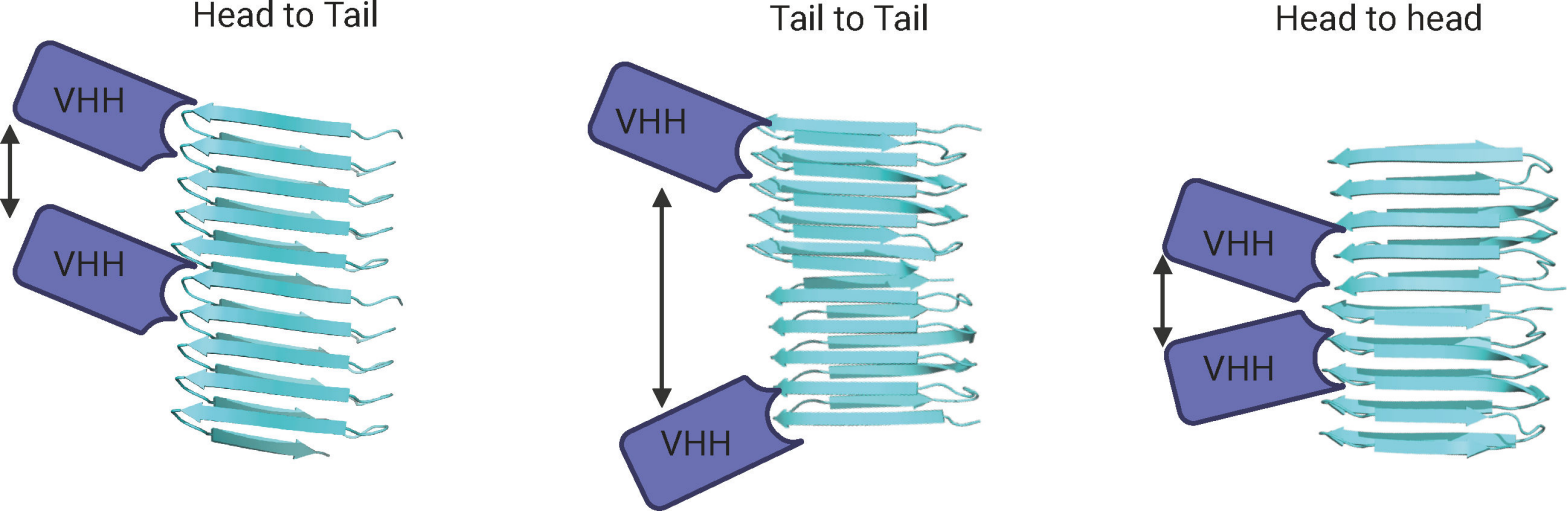
Using a nanobody to obtain the high-resolution structure of CsgA fibrils and to localize N/C regions of CsgA units in the filament (created in https://BioRender.com).

We also see this response as an opportunity to highlight two potential strategies we have considered for achieving a higher resolution cryo-EM structure and, ultimately, resolving the monomer connectivity in CsgA amyloid fibrils. First, we were particularly inspired by the study by Deng et al. (9) on the structure of bactofilin filaments, where they used nanobodies to determine the orientation of monomers within the filaments. We believe a similar approach could be applied to CsgA by immunizing alpacas with CsgA amyloids and isolating nanobodies that specifically bind to a defined structural epitope. Such nanobodies could aid in the localization of the monomer orientation within the fibril and may also help stabilize the complex and de-bundle CsgA fibrils, potentially enabling higher resolution structures to be achieved (Fig. 1).

**Fig 2:**
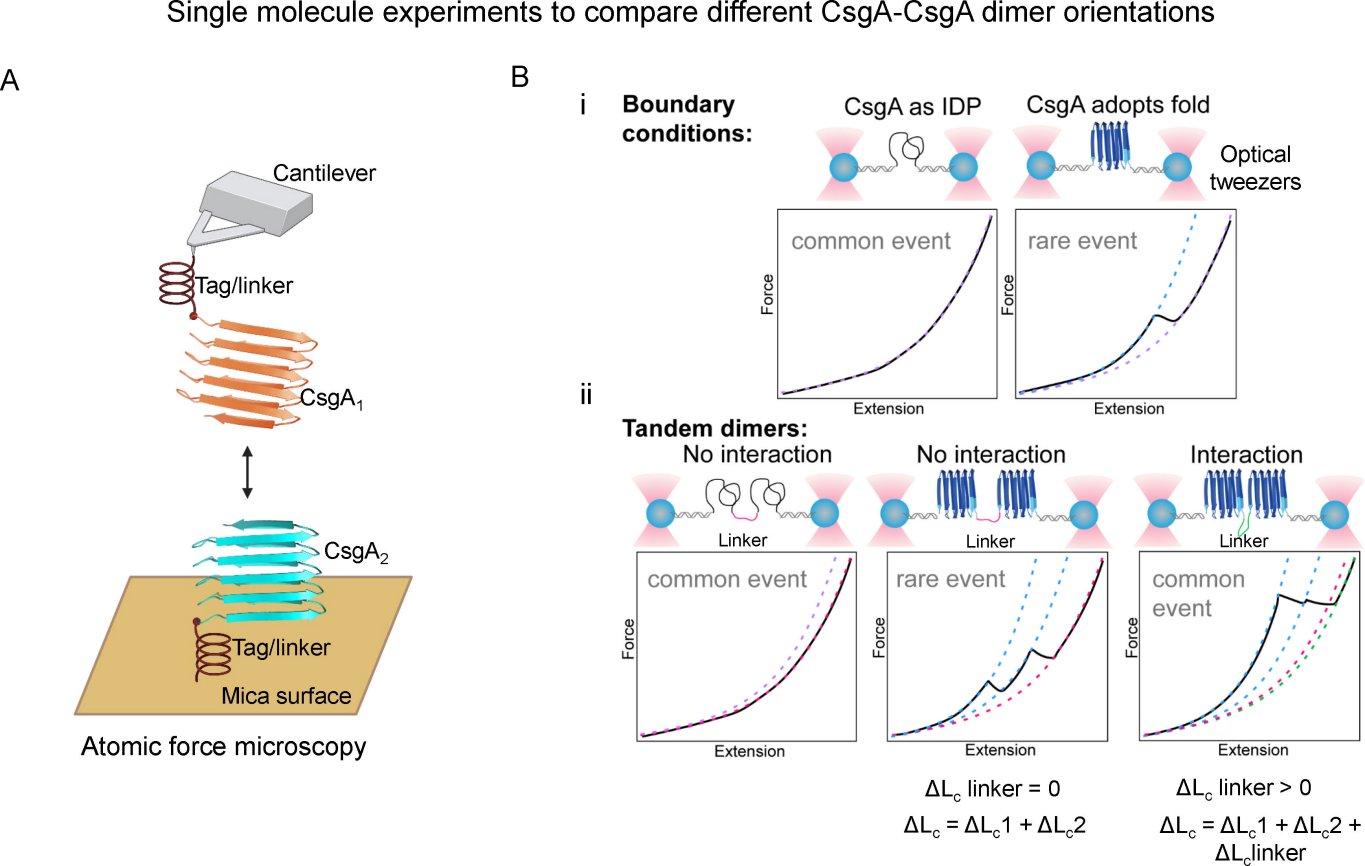
Single molecule force spectroscopy experiments to experimentally uncover the preferred arrangement of CsgA dimers through atomic force microscopy (**A**) and optical tweezers (**B**). CsgA, when tethered between DNA handles and trapped beads, may exist in an unstructured state with no unfolding transition, or in a folded state that exhibits an unfolding transition. Partially folded states are also possible (i). For the CsgA tandem dimer in different orientations (NC-NC, CN-NC, or NC-CN), we may observe three different scenarios (ii): first, the dimer may be unstructured (ii, left); second, the two monomers may not interact (ii, center), in which case a total contour length change of ΔLc1 + ΔLc2 will be recorded; third, one CsgA molecule may interact with another in a specific orientation (ii, right), leading to a total contour length change of ΔLc1 + ΔLc2 + ΔLclinker, corresponding to the two CsgA units plus the flexible linker. Furthermore, owing to the added stability, both the unfolding forces and the frequency of observing unfolding events will be higher. Dashed lines indicate worm-like-chain models that describe stretching of the DNA handles and unfolded protein (created in https://BioRender.com).

Second, we recognize that cryo-EM data processing often involves averaging effects, which can obscure less dominant fibril arrangements when particle numbers are insufficient for reliable three-dimensional reconstruction. To address this, Derek Dee’s group is currently exploring CsgA-CsgA interactions at the single-molecule level using optical tweezers (Fig. 2). By recording force-extension curves for different CsgA dimer orientations: head-to-head, head-to-tail, or tail-to-tail, we aim to experimentally determine whether specific configurations of CsgA dimers are present.

Comparing tandem dimers NC-NC vs CN-NC and NC-CN, we can verify the directionality of CsgA amyloid growth (Fig. 2B, ii). If amyloid elongation proceeds in the NC-NC orientation, we expect a total contour length change (ΔLc) corresponding to the unfolding of two CsgA units plus the length of the flexible linker connecting them (e.g., in an NC-linker-NC construct) (Fig. 2B, ii, right panel). In contrast, if CsgA dimers do not form head-to-head or tail-to-tail interactions, then pulling on CN-NC or NC-CN constructs should result in a ΔLc reflecting only the two individual CsgA units, excluding the linker (Fig. 2B, ii, center panel). This would suggest that the units behave as separate, non-interacting domains during unfolding. Moreover, if the two CsgA units unfold and refold independently, their thermodynamic and kinetic signatures will differ from those of a cooperatively interacting dimer. A more stable dimer is more likely to exhibit an unfolding transition in force-extension curves, in contrast to the unstructured, featureless curves typically observed for monomeric CsgA (Fig. 2B, i). These differences can help distinguish between interacting versus non-interacting CsgA configurations. Therefore, this complementary single-molecule approach offers a promising avenue for uncovering the potential structural heterogeneity.

### Concluding remarks

We want to clearly state that our goal has never been to mislead or obscure the field, nor to challenge the authority of those who have made foundational contributions. Rather, our intention is to advance the field by making a small but meaningful step forward and contributing intellectually to the ongoing exploration of CsgA amyloid structures.

To support future research in this area, we have deposited our original raw, non-motion-corrected movies in the Electron Microscopy Public Image Archive (accession number: EMPIAR-12788). We welcome collaborations with structural biologists who specialize in filamentous systems to further analyze these data using advanced image processing techniques. We hope that experts in the field will recognize and appreciate our initial efforts, even as ongoing technological advancements enable more detailed and refined analyses. We value open-minded and collaborative approaches that can help drive this work/research area forward.

We deeply acknowledge and respect the pioneers in this area, whose groundbreaking work has provided the essential foundation that enabled our progress on this project. We sincerely encourage readers to read the full paper, where we believe valuable insights can be found, and to remain both critical and creative in their thinking. Solving the structural and mechanistic questions surrounding CsgA amyloids will undoubtedly require continued innovation, open collaboration, and increasingly sophisticated approaches in the future.
